# An exploration of sub-national variability in institutional maternal mortality ratios in Kenya: a meta-analysis of the 2021 health facility data

**DOI:** 10.3389/fgwh.2025.1481495

**Published:** 2025-03-04

**Authors:** Francis G. Muriithi, Christina Easter, Alfred Osoti, Zahida Qureshi, Adam Devall, Arri Coomarasamy

**Affiliations:** ^1^ WHO Collaborating Centre for Global Women's Health, Department of Metabolism and Systems Science, School of Medical Sciences, College of Medicine and Health, University of Birmingham, Birmingham, United Kingdom; ^2^ Institute of Applied Health Research, College of Medical and Dental Sciences, Public Health Building, University of Birmingham, Birmingham, United Kingdom; ^3^ Department of Obstetrics and Gynaecology, School of Medicine, College of Health Sciences, University of Nairobi, Nairobi, Kenya

**Keywords:** maternal mortality, sub-Saharan Africa, Kenya, sub-national, variability, positive deviance

## Abstract

**Background:**

In many countries in sub-Saharan Africa, the burden of preventable maternal deaths is still unacceptably high. Most Maternal Mortality Ratio (MMR) estimates are national, rarely sub-national. This study explores Kenya's 2021 national health facility dataset on maternal deaths and live births for sub-national variability to describe the pattern and trend in variation in institutional maternal mortality ratios (iMMRs).

**Methods:**

Country-wide health facility data on live births and maternal deaths for 2021 were requested from the District Health Information System (DHIS-2). A descriptive comparison of sub-national (Regional and County) iMMRs to national iMMR was carried out. Against a national average iMMR for Kenya of about 100 per 100,000 live births, those regions and counties with an iMMR <75 per 100,000 live births were defined as positive outliers, and those with an iMMR >125 were defined as negative outliers.

**Results:**

In 2021, 1,162 maternal deaths and 1,174,774 live births occurred within Kenya's health facilities. The annual national average iMMR was 99 per 100,000 live births [95% confidence interval (CI): 93.3, 104.8]. There was sub-national variability in iMMR at both regional and county levels. Central, Western and Rift Valley regions were positive outliers; North-Eastern Coast and Nairobi regions were negative outliers, while Nyanza and Eastern regions had an iMMR consistent with the national average. Seventeen counties were positive outliers, namely Baringo, Siaya, Nyamira, Elgeyo-Marakwet, West Pokot, Nandi, Kiambu, Laikipia, Nyeri, Samburu, Marsabit, Vihiga, Bungoma, Nyandarua, Kajiado, Murang'a and Trans-Nzoia. Ten counties were negative outliers: Tana River, Mandera, Machakos, Kilifi, Taita–Taveta, Kisumu, Nairobi, Garissa, and Mombasa and Isiolo. The iMMR in the remaining twenty counties was consistent with the national average. The effect sizes of the observed health facility variation were zero and there was no evidence of month-to-month variation.

**Conclusion:**

There is evidence of sub-national variability in Kenya's iMMRs. Understanding these reasons for the variability is crucial for developing strategies for improving maternal health outcomes. If positively deviant behaviours and practices are identified, they could form the basis for adopting asset-based approaches such as the positive deviance approach to improve maternal healthcare delivery processes and outcomes and reduce preventable maternal deaths.

## Introduction

In many sub-Saharan African countries, the burden of preventable maternal deaths is still unacceptably high. Recent evidence shows that sub-Saharan Africa (SSA) contributed to 70% of global maternal deaths in 2020 and that SSA's average Maternal Mortality Ratio (MMR) of 536 per 100,000 live births [Uncertainty Interval (UI) of 469–640] is more than twice the global average MMR of 223 per 100,000 live births (UI: 202–255) (1). As a result of this high burden of maternal deaths, the surviving children face adverse health and socioeconomic outcomes, often lasting more than one generation (2, 3). Therefore, eliminating preventable maternal deaths has the potential to safeguard the health and socioeconomic outcomes of families that would struggle to thrive without a mother.

To successfully implement a strategy for eliminating preventable maternal deaths, it is crucial to establish accurate metrics on the number of women dying during and after childbirth, including where and why they died (4). Global estimates of country-level maternal mortality ratios (MMRs) are typically calculated by international agencies such as the United Nations using multiple sources of data on maternal deaths that occur in both community and health facility settings (1, 5). While these country MMR estimates help inform the global agenda, tracking progress and comparison between countries, their utility at the sub-national level within individual countries may be limited (6). In addition, global estimates lack granularity regarding which deaths occur in the community vs. those at health facilities (7). The paucity of insight into sub-national data and where deaths occur may hinder the focused and contextualised approach to tackling the persistent problem of preventable maternal mortality in SSA (8).

To fill this void, models such as the Demographic and Health Survey (DHS) that use data from national surveys to estimate sub-national MMR estimates have been proposed (9, 10). Maternal mortality data reported directly from health facilities have occasionally been utilised to compute MMR estimates. However, there are concerns regarding selection bias compared to other data sources and that institutional data reflects only those women who can access health facility care (11, 12). Despite these concerns, institutional data are highly specific to local contextualised conditions and thus better-suited to inform local policymakers, clinicians and programs for strengthening health facilities and the health system (12). Institutional data, such as iMMR, can offer valuable insights into how delays in receiving appropriate care at a health facility (third delay) contribute to maternal deaths and inform quality improvement within health systems (13, 14).

Health systems in low- and middle-income countries have made significant progress in having women deliver under skilled birth attendants (15). For example, in Kenya, as per the 2022 Kenya Demographic and Health Survey (KDHS) report, nearly 90% of women deliver under skilled birth attendants (16). Delivery under the care of skilled birth attendants is associated with a significant reduction in maternal mortality (17). Countries with high maternal mortality rates often have low coverage of skilled birth attendance, whereas countries with low maternal mortality rates tend to have high coverage of skilled birth attendance (18). Despite this achievement in the proportions of women delivering under skilled birth attendants, some countries, such as Kenya, paradoxically continue to exhibit high maternal mortality ratios (19). One explanation is that skilled birth attendants require an enabling work environment for their presence at birth to translate into quality care and a reduction in preventable morbidity and mortality (19–21). An enabling work environment for skilled birth attendants has been described as one that provides necessary inputs such as supportive regulations, policies, infrastructure, communication, referral systems, logistics, and supplies (19, 22).

In Kenya specifically, evidence from a confidential enquiry into maternal deaths showed a need for quality improvement at the health facility level to avert preventable maternal deaths (23). Specific health facility-level recommendations included Regular and mandatory healthcare worker updates in emergency obstetric care, increased availability and safety of blood and blood products, improved monitoring during the antenatal, intrapartum and postpartum periods, especially for women with high-risk pregnancies, safer anaesthesia, audit and feedback processes and improved documentation (23, 24).Following documentation at the health facility level, health data on key processes and outcomes, including maternal deaths in Kenya, are reported through the District Health Information System Software-2 (DHIS-2) for aggregation nationally (25, 26). Since the enactment of a new constitution in 2010, healthcare delivery has devolved from the national government to 47 sub-national governments referred to as counties (27, 28). Health service delivery is organised into four levels: Community services, primary health services, county referral services and national referral services (28). The health facilities that serve these four levels are categorised into six categories: Level 1 (Community health facility), Level 2 (Clinics and dispensaries), Level 3 (Health centres and nursing and maternity homes), Level 4 (County Hospitals), Level 5 (County referral hospitals) and Level 6 (National referral, teaching and specialised hospitals) (29). All these levels have a role to play in the provision of maternal health care in Kenya.

We postulated that disaggregation of national MMR estimates into sub-national levels might reveal variability between regions and health facilities and facilitate the identification of the most appropriate approaches for improving maternal health outcomes and, by extension, tackling the causes of preventable maternal deaths.

While progress in health indices of most countries, including maternal mortality, is monitored at the national level, there is potential benefit in exploring variability within countries (sub-national level) for the purpose of ensuring equity in the distribution and use of health resources and tracking progress (8). Learning from sub-national variability may also provide unique behavioural and practice insights from those accessing and/or delivering maternal healthcare (30). Variability is defined by assessing the performance of an entity, such as a health facility, in relation to an expected performance or set target (31). The terms used to describe variability include high or low, positive or negative outlier or positive or negative deviance depending on the extent of deviation from the expected performance or set target (31–33). Unique behavioural and practice insights from positive outlier regions or health facilities may help the negative outliers to solve shared challenges with the overall effect of improving maternal health outcomes through a positive deviance approach (30, 34–36). Such a shared challenge may be maternal mortality, with positive outlier regions, counties and health facilities providing insights for improving the negative outlier ones.

This study explores Kenya's 2021 national health facility dataset on maternal deaths and live births for sub-national variability to describe the pattern and trend of variation in Institutional Maternal Mortality Ratios (iMMRs). We posed the following three research questions (RQ): (1) What is the pattern of sub-national variation in iMMR in Kenya? (2) What is the pattern of iMMR variation by health facility level? and (3) What is the pattern of month-to-month county iMMR variation?

## Methods

The research protocol for this work was reviewed by the University of Birmingham (Reference ERN_22-0550) and the Kenyatta National Hospital—University of Nairobi Ethics and Research Committee (ERC) (Reference KNH-ERC/RR/712). This work was conducted under the National Commission for Science, Technology, and Innovation (NACOSTI) license number NACOSTI/P/22/20651.

In October 2022, we submitted a DHIS2 data access request to the Division of Research and Development team via the office of the Director General, Ministry of Health, Kenya. The data access was authorised under reference MOH/ADM/1/1/82 (263).

The specific datasets requested for this project included: Healthcare facility by name and level, the total number of maternal deaths per facility for 12 months by facility (the latest complete annual dataset) and the total number of live births per facility for the same period as the maternal deaths.

### Data preparation

After exploring the data for completeness, we categorised the health facilities by county using the Ministry of Health facility list and categories (Level 1 to Level 6). Kenya operates two levels of Government—national and county with the latter being the most relevant for healthcare system administration as it is a devolved function (37). We then collated the total institutional maternal deaths and live births per health facility, which we aggregated by county and region. iMMR was computed as (total maternal deaths/total live births) *100,000 live births.

### Statistical analysis

Data analysis was performed using Stata statistical software, release 17 (StataCorp. LLC, College Station, TX, USA). We calculated and presented graphically the 95% Confidence Interval (CI) for each of the county and regional iMMR values, conducted a meta-analysis of iMMRs by health facility level, and compared mean monthly iMMRs. Standard STATA statistical commands were utilized and are available upon request to the corresponding author.

### Outlier identification

Although a range of statistical and non-statistical methods have been proposed for outlier identification, there is no defined gold-standard method (31, 33, 38, 39). In our study, using the national average iMMR as the reference, we defined a positive outlier as when an iMMR was 25 centiles less than the national iMMR and a negative outlier as when an iMMR was 25 centiles greater than the national iMMR. iMMRs falling in between the two cut-offs were determined to be consistent with the national average.

Against a national average iMMR for Kenya of around 100 per 100,000 live births, those regions, or counties with an iMMR <75 per 100,000 live births were defined as positive outliers, and those with an iMMR >125 were defined as negative outliers.

We did not conduct a health facility-to-health facility level analysis. The reasoning behind this decision was that maternal deaths are few between facilities, making it more likely to identify the mothers who died, leading to a breach of confidentiality. This approach is similar to that of the Centres for Disease Control and Prevention (CDC), which advises against presenting or publishing death counts or rates of nine or fewer for sub-national geography (40). In addition, few numbers of maternal deaths may lead to small sample size bias, statistical imprecision and flawed inferences (41).

## Results

We analysed 1,162 maternal deaths, and 1,174,774 live births reported to the DHIS2 from facilities where maternal deaths occurred in 2021. The health facilities consisted of private, faith-based/mission, and public health facilities across all the 47 counties of Kenya. A total of 323 health facilities were distributed as 49 level 2, 53 level 3, 197 level 4, 19 level 5, and 5 level 6 health facilities.

We present the results of our analysis of sub-national variation in iMMR below in four parts: Regional, county, health facility, and month-to-month variation.

### Regional level variation

The computed regional institutional maternal mortality ratios (iMMRs) and the corresponding 95% confidence intervals are presented in Table 1.

**Table 1:** Computed regional iMMR (95% CI) estimates.

Region	Institutional maternal deaths (*n*)	Institutional live births (*n*)	iMMR (95% CI)
Coast	172	116,768	147.3 (126.1,171.0)
North Eastern	83	56,380	147.2 (117.3,182.5)
Eastern	142	136,688	103.9 (87.5,122.4)
Central	72	122,968	58.6 (45.8,73.7)
Rift Valley	236	315,172	74.9 (65.6,85.1)
Western	94	127,371	73.8 (59.6,90.3)
Nyanza	159	173,862	91.5 (77.8,106.8)
Nairobi	204	125,565	162.5 (141.0,186.3)
National	1,162	1,174,774	98.9 (93.3,104.8)

**Figure 1:**
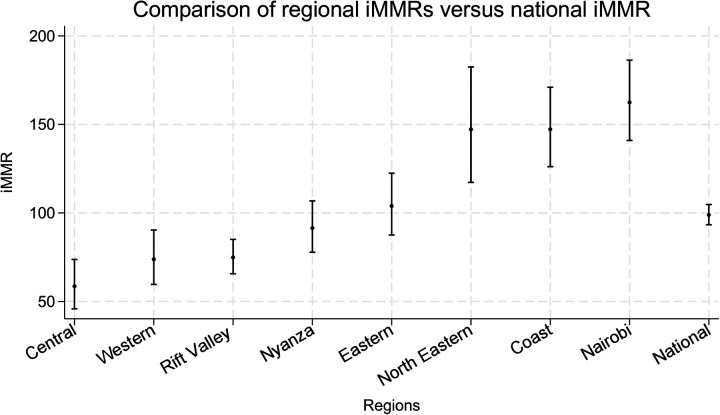
Variation in sub-national iMMR at the regional level. National and regional areas of Kenya displayed graphically providing the iMMR values (dots) and corresponding 95% confidence intervals (interval lines around the iMMR dots).

Overall, we found that there was regional variation in iMMR**.** The region that had the lowest iMMR was Central Kenya at 58.6 (95% CI 45.8–73.7), and the highest was Nairobi at 162.5 (95% CI 141.0–186.3). Based on the defined cut-offs, Central 58.6 (95% CI: 45.8, 73.7), Western 73.8 (95% CI: 59.6, 90.3), and Rift Valley 74.9 (95% CI: 65.6, 85.1) regions were positive outliers; and North-Eastern 147.2 (95% CI: 117.3, 182.5), Coast 147.3 (95% CI: 126.1, 171.0) and Nairobi 162.5 (95% CI: 141.0, 186.3) were negative outliers, while Nyanza 91.5 (95% CI: 77.8, 106.8) and Eastern regions 103.9 (95% CI: 87.5, 122.4) had iMMR consistent with the national average (Table 1, Figure 1).

### County-level variation

The computed county institutional maternal mortality ratios (iMMRs) and the corresponding 95% confidence intervals are presented in Table 2.

**Table 2:** Computed county iMMR (95% CI) estimates.

County code	County	Institutional maternal deaths (n)	Institutional live births (n)	iMMR (95% CI)
001	Mombasa	62	31,243	198.4 (152.2,254.3)
002	Kwale	23	23,485	97.9 (62.1,146.9)
003	Kilifi	62	42,817	144.8 (111.0,185.6)
004	Tana River	10	7,300	137.0 (65.7,251.8)
005	Lamu	4	4,404	90.8 (24.8, 232.4)
006	Taita-Taveta	11	7,519	146.3 (73.1,261.6)
007	Garissa	43	23,419	183.6 (132.9,247.2)
008	Wajir	11	11,851	92.8 (46.3, 166.0)
009	Mandera	29	21,110	137.4 (92.0, 197.2)
010	Marsabit	5	9,269	53.9 (17.5,125.8)
011	Isiolo	12	6,026	199.1 (102.9, 347.6)
012	Meru	29	31,265	92.8 (62.1, 133.2)
013	Tharaka-Nithi	12	9,618	124.8 (64.5, 217.8)
014	Embu	10	12,003	83.3 (40.0, 153.2)
015	Kitui	17	21,515	79.0 (46.0, 126.5)
016	Machakos	38	26,773	141.9 (100.5, 194.8)
017	Makueni	19	20,219	94.0 (56.6, 146.7)
018	Nyandarua	7	11,152	62.8 (25.2,129.3)
019	Nyeri	8	16,502	48.5 (20.9,95.5)
020	Kirinyaga	15	13,359	112.3 (62.9,185.1)
021	Murang'a	13	19,457	66.8 (35.6,114.2)
022	Kiambu	29	62,498	46.4 (31.1,66.6)
023	Turkana	27	23,492	114.9 (75.8,167.2)
024	West Pokot	8	18,209	43.9 (19.0,86.6)
025	Samburu	4	8,004	50.0 (13.6, 127.9)
026	Trans-Nzoia	15	21,077	71.2 (39.8,117.4)
027	Uasin Gishu	28	30,441	92.0 (61.1, 132.9)
028	Elgeyo-Marakwet	4	11,484	34.8 (9.5,89.2)
029	Nandi	6	16,253	36.9 (13.6,80.3)
030	Baringo	3	13,393	22.4 (4.6,65.5)
031	Laikipia	8	16,926	47.3 (20.4,93.1)
032	Nakuru	49	59,819	81.9 (60.6,108.3)
033	Narok	24	24,129	99.5 (63.7, 148.0)
034	Kajiado	19	29,368	64.7 (39.0,101.0)
035	Kericho	23	22,574	101.9 (64.6, 152.8)
036	Bomet	18	20,003	90.0 (53.3,142.2)
037	Kakamega	37	45,123	82.0 (57.7, 113.0)
038	Vihiga	7	11,912	58.8 (23.6, 121.0)
039	Bungoma	31	51,074	60.7 (41.2, 86.1)
040	Busia	19	19,262	98.6 (59.4, 154.0)
041	Siaya	9	27,538	32.7 (15.0, 62.0)
042	Kisumu	53	33,356	158.9 (119.0, 207.8)
043	Homa Bay	25	27,962	89.4 (57.9, 132.0)
044	Migori	32	39,102	81.8 (56.0, 115.5)
045	Kisii	35	30,885	113.3 (79.0, 157.6)
046	Nyamira	5	15,019	33.3 (10.8, 77.7)
047	Nairobi	204	125,565	162.5 (141.0, 186.3)
n/a	National	1,162	1,174,774	98.9 (93.3, 104.8)

Overall, we found that there was variation in county-level iMMR. The lowest iMMR was Baringo at 22 1(95% CI: 4.6–65.5), and the highest was Isiolo at 199 1(95% CI: 79.7–105.8).

Based these cut-offs, seventeen counties, including Baringo, Siaya, Nyamira, Elgeyo-Marakwet, West Pokot, Nandi, Kiambu, Laikipia, Nyeri, Samburu, Marsabit, Vihiga, and Bungoma, Nyandarua, Kajiado, Murang'a and Trans-Nzoia were determined to be positive outliers, with their iMMR ranging from 22.4 [95% CI: (4.6, 65.5) to 71.2 60.7 (95% CI: 39.8, 117.4) (41.2, 86.1)]. Ten counties, including Tana River, Mandera, Machakos, Kilifi, Taita—Taveta, Kisumu, Nairobi, Garissa, and Mombasa and Isiolo, were determined to be negative outliers, with their iMMR ranging between 137.044.8 (95% CI: 65.7111, 0.251.8185.6) and 199.18.4 (95% CI: 102.952.2, 347.6254.3). The remaining 20 counties were found to have an iMMR consistent with the national average. These counties were Kwale, Lamu, Wajir, Meru, Tharaka—Nithi, Embu, Kitui, Makueni, Kirinyaga, Turkana, Uasin Gishu, Nakuru, Narok, Kericho, Bomet, Kakamega, Busia, Homa Bay, Migori and Kisii.

**Figure 2:**
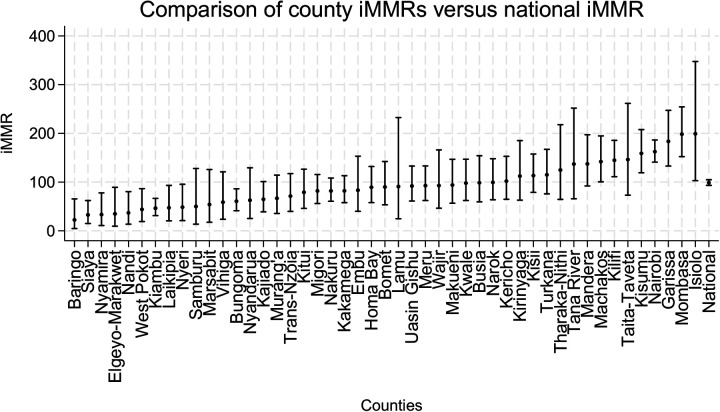
Variation in sub-national iMMR at the county level. National and county areas of Kenya are represented in the graph providing the iMMR values (dots) and corresponding 95% confidence intervals (interval lines around the iMMR dots).

The graphical representation of regional iMMRs vs. National iMMR is in Figure 2.

### Health facility level variation

Overall, we found that there was iMMR variation by health facility level. Level 6 and level 2 health facilities contributed the greatest proportion of institutional maternal deaths. Although there was variation in the relative contribution of various levels, the effect sizes were zero, implying limited practical application of this finding.

**Figure 3:**
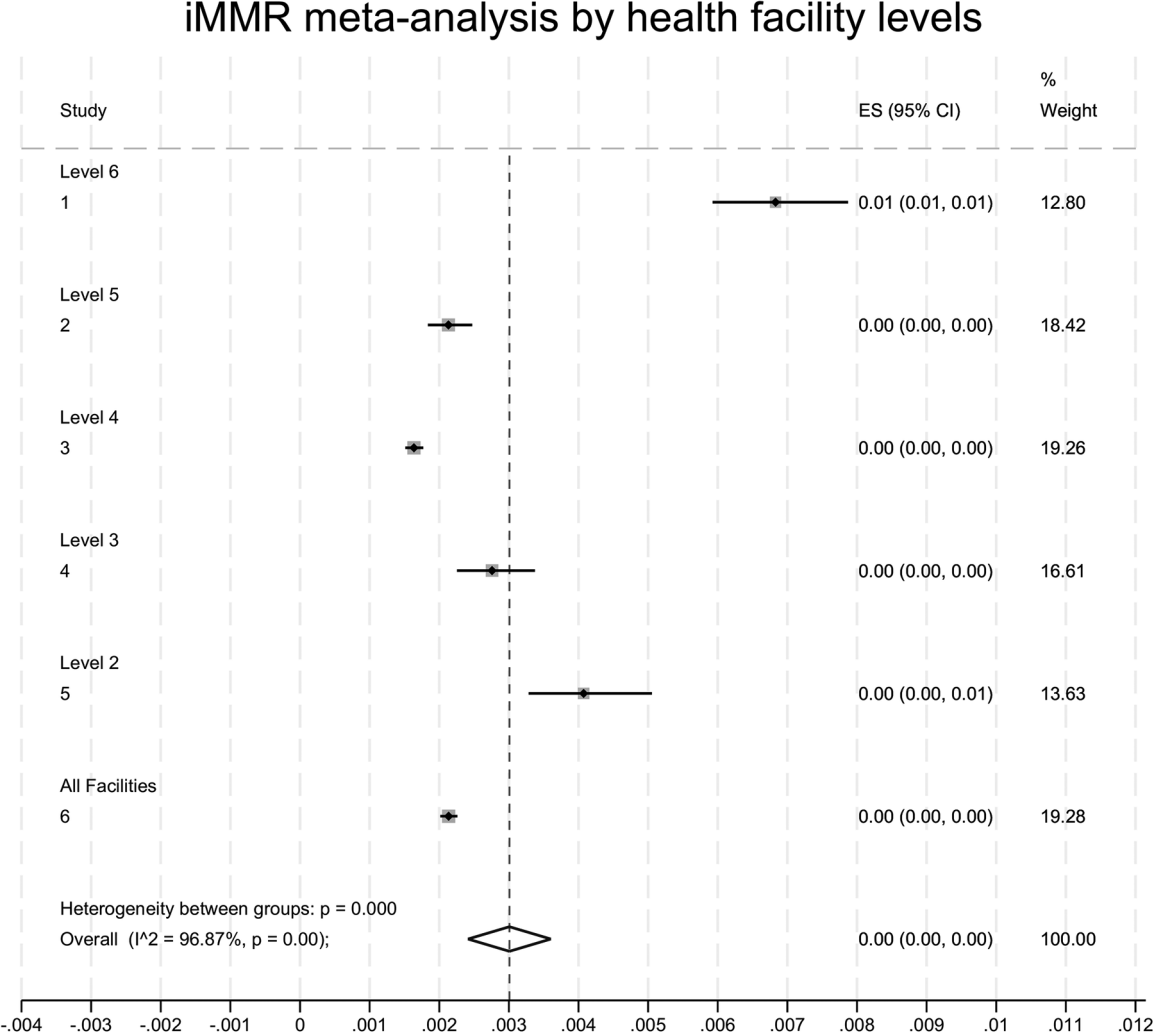
Meta-analysis of institutional maternal mortality ratios (iMMRs) and 95% CI for the level of facilities. Level 1 is a community health facility. Level 2 is dispensaries and private clinics. Level 3 is health centres. Level 4 is sub-county hospitals and nursing homes. Level 5 is County referral hospitals, teaching and referral hospitals, and Level 6 is the National Referral Hospital.

**Note:** There were no maternal deaths reported from level 1 facilities. The live births figure at county and regional levels (*n* = 1,174,774) differs from the figure above (*n* = 544, 239) because it includes births that occurred at all facilities (those that reported maternal deaths and those that did not).

These findings are summarised in Table 3 and the meta-analysis presented in Figure 3.

**Table 3:** Computed health facility level iMMR (95% CI) estimates.

Level of health facility^a^	Institutional maternal deaths (*n*)	Institutional live births (*n*)	iMMR (95% CI)
Level 6	189	27,671	683.0 (589.4, 787.2)
Level 5	173	81,132	213.2 (182.7, 247.4)
Level 4	624	381,233	163.7 (151.1, 177.03)
Level 3	94	34,076	275.9 (223.0, 337.5)
Level 2	82	20,127	407.4 (324.2, 505.5)
Level 1	0	-	—
All facilities	1,162	544,239	213.5 (201.4, 226.1)

^a^ Level 1 (Community health facility), Level 2 (Clinics and dispensaries), Level 3 (Health centres and nursing and maternity homes), Level 4 (County Hospitals), Level 5 (County referral hospitals) and Level 6 (National referral, teaching and specialised hospitals).

### Month to month iMMR variation

**Figure 4:**
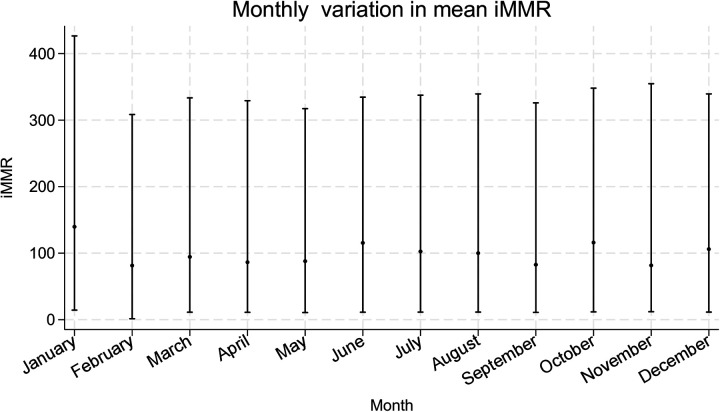
Month-to-month variation in mean iMMRs. National monthly iMMRs for Kenya in the year 2021 are represented in the graph providing the iMMR values (dots) and corresponding 95% confidence intervals (interval lines around the iMMR dots).

Overall, there was no evidence of month-to-month variation in iMMR. The findings are illustrated in Figure 4.

## Discussion

We aimed to explore Kenya's institutional maternal mortality ratios (iMMRs) for sub-national variability. We conducted our analysis at the regional, county and aggregated health facilities by level avoiding individual health facility comparisons. This approach in the analysis was because healthcare governance and implementation are devolved functions (37). We avoided individual health facility comparisons as a good practice to minimise the risk of breach of confidentiality, especially at health facilities that had few maternal deaths (4, 40). We felt that such an analysis would have required consent or ethical approval by each of the participating health facilities.

Our analysis of the 2021 maternal deaths and live births data for Kenya established the following key findings: (1) there is evidence of sub-national variability in institutional maternal mortality ratios at regional and county levels; (2) here is evidence of variation in the relative contribution to the overall iMMRs by the various levels of health facilities. However, the effect size was zero, implying limited practical application of this finding; (3) the monthly mean iMMR trend did not reveal any variation.

To contextualise, Kenya has had two Confidential Enquiry into Maternal Deaths (CEMD) reports on deaths between 2014 and 2016 (23, 24). The analysis in both reports hints about the presence of regional variation, with most maternal deaths occurring within the Rift Valley, Western and Coast regions (23). Regional administrative boundaries have been historical since the promulgation of Kenya's new constitution in 2010, when healthcare governance was devolved into the county-level (37). Therefore, a county-by-county analysis would have provided more insight into the pattern and causes of sub-national variability.

Despite this, there were commonalities in both CEMD reports regarding the most frequent causes of death and the association of maternal deaths with healthcare worker-related factors (23, 24). The leading causes of maternal deaths nationally were obstetric haemorrhage (39.7%), hypertensive disorders (15.3%), and non-obstetric complications (19.8%) (23, 24). The healthcare worker-related factors included delays in starting treatment (32.9%), inadequate monitoring (26.9%), inadequate clinical skills (28.1%), prolonged abnormal observation without action (23.6%), and incomplete initial assessment (22.7%) (7, 24). Therefore, based on the CEMD evidence, we postulate that a possible explanation for the sub-national variability in iMMR is differences in healthcare worker-related factors within the health facilities in the various counties.

The reasons for the regional and county level variability in iMMRs in Kenya could not be determined using the provided data. Existing literature acknowledges diversity in maternal health indices such as maternal deaths and cites various explanatory reasons for such variability. These reasons include sub-national variations in political commitment to reducing maternal mortality, health system status and resilience, inequitable access to healthcare, poverty, and education (42–45). Temporal variability in maternal mortality may also be influenced by seasonal factors such as weather and the burden of seasonal infectious diseases like malaria, political instability, conflict, and healthcare worker strikes (46–48). Factors within health facilities include variations in service delivery such as triage, monitoring, and referral; availability of life-saving medications, equipment and blood transfusion; and staffing-related issues such as absence, competence and supervision of juniors (20, 23, 24). There is a need to further examine and compare Kenya's counties to explain the spatial and temporal variability in iMMRs and their potential application to tackling the causes and factors contributing to maternal mortality within counties.

Regarding the observed variation between various levels of health facilities, we postulate that this may reflect the status referral system where complicated cases are referred upwards and often arrive late for the case of level 6 health facilities. On the other hand, level 2 facilities are dispensaries. Although the dispensaries are closest to the community, they may be ill-equipped to handle even basic obstetric emergencies. The maternal healthcare referral system may cushion level 4 and 5 health facilities because they can refer complex cases upwards to level 6 facilities. Our meta-analysis revealed no effect size, implying that the variations in iMMR at the health facility level are likely to be of limited practical application.

A recent study that analysed pregnancy outcomes in health facility deliveries in Kenya and Uganda found that more pre-discharge deaths occurred after a maternal referral and following a caesarean section (49). Referred mothers are especially at risk because of sub-optimal care in ill-equipped lower-level health facilities and bottlenecks within the referral pathway (50).

Finally, the lack of month-to-month variation was unexpected, given evidence from Mozambique and Burkina Faso linking seasonal iMMR fluctuations to income declines, healthcare access barriers, and malaria-related maternal deaths (46, 51, 52). We did not find published work examining seasonality and maternal mortality in Kenya. We postulate that our finding of the absence of month-to-month variation may be due to the low number of maternal deaths per month or the limited one-year study period.

### Strengths and weaknesses

The use of data from DHIS2 could be both a strength and a weakness. A strength in that this approach minimises the potential hawthorne effect if we were to collect these data directly from the healthcare facilities (53). We acknowledge the limitations in using such an approach as the quality and completeness of data are not directly verifiable at the point of the event and data entry (23). The subject of maternal death is prone to “shame” and “blame” at the health facility level, further increasing the risk of underreporting and selective reporting due to fear of the consequences (54). In addition, research that utilises secondary data is prone to researcher bias due to its exploratory nature (55).

While DHIS2 is a pragmatic source of health facility data, studies from Kenya, South Africa and Nigeria, have highlighted its limitations such as incomplete and inconsistent reporting across facilities and data duplication (56–59). We postulate that one possible source of duplication and inconsistency is when a mother is referred from one facility and dies en route to another, with both facilities potentially reporting her data to DHIS2—It was unclear to us how such cases were managed. Therefore, our findings should be interpreted with the awareness of these data quality concerns especially since it was not possible for us to authenticate data accuracy from its input at the health facility or its processing at the Ministry of Health.

### Suggestions for future research

Future research should consider exploring the explanations of sub-national variation in iMMR, especially between health facilities that are near similar levels. Ideally, they should identify shared challenges and unique practices and behaviours that may explain variations where challenges are similar.

In addition, future research should consider comparing iMMR between public, private, and faith-based/mission hospitals and explanations for any observed differences. Such a comparison could offer unique insights into how different hospitals address shared challenges, creating an avenue for collaborative improvement in maternal health outcomes. Consent or ethical approval from the participating health facilities would be necessary as there is potential breach of confidentiality where the maternal deaths are in single digits.

Additionally, future studies should explore linking health facility data with geographical and socio-demographic determinants within their catchment areas to enhance the understanding of factors contributing to the observed sub-national variation in iMMR. Data from the national census or demographic and health surveys could be considered for that linkage. Future research on seasonal variation in maternal mortality should compare data on similar months or seasons over two or more years, not a single year.

Finally, research is required to improve the quality of health facility data reporting and processing and quality control mechanisms.

## Conclusion

There is evidence of sub-national variability in Kenya's iMMRs at regional and county levels and between various health facility levels in the referral system. This variability presents an opportunity to explore the reasons for any observed positive and negative variability. Understanding these reasons is crucial for developing alternative and targeted strategies for improving maternal health outcomes. If positively deviant behaviours and practices are identified, they could form the basis for adopting asset-based approaches such as the positive deviance approach to improve maternal healthcare delivery processes and outcomes and reduce preventable maternal deaths.

## Data Availability

The data analyzed in this study is subject to the following licenses/restrictions: The data set used in this study contains individual health facility data. These data are available from the Division of Research & Innovation, Ministry of Health, Kenya, upon application and approval by the Director General of Health. Requests to access these datasets should be directed to Head of Division of Research and Innovation via the Director General, Ministry of Health, Kenya. Afya House, Cathedral Road P.O Box 30016—00100 Nairobi, Kenya https://www.health.go.ke/contact-us.
